# Probing the Activity Modification Space of the Cysteine Peptidase Cathepsin K with Novel Allosteric Modifiers

**DOI:** 10.1371/journal.pone.0106642

**Published:** 2014-09-03

**Authors:** Marko Novinec, Brigita Lenarčič, Antonio Baici

**Affiliations:** 1 Department of Chemistry and Biochemistry, Faculty of Chemistry and Chemical Technology, University of Ljubljana, Ljubljana, Slovenia; 2 Department of Biochemistry, University of Zürich, Zürich, Switzerland; 3 Department of Biochemistry and Molecular and Structural Biology, Jožef Stefan Institute, Ljubljana, Slovenia; Stanford University, United States of America

## Abstract

Targeting allosteric sites is gaining increasing recognition as a strategy for modulating the activity of enzymes, especially in drug design. Here we investigate the mechanisms of allosteric regulation of cathepsin K as a representative of cysteine cathepsins and a promising drug target for the treatment of osteoporosis. Eight novel modifiers are identified by computational targeting of predicted allosteric sites on the surface of the enzyme. All act via hyperbolic kinetic mechanisms in presence of low molecular mass substrates, as expected for allosteric effectors. Two compounds have sizable effects on enzyme activity using interstitial collagen as a natural substrate of cathepsin K and four compounds show a significantly stabilizing effect on cathepsin K. The concept of activity modification space is introduced to obtain a global perspective of the effects elicited by the modifiers. Analysis of the activity modification space reveals that the activity of cathepsin K is regulated via multiple, different allosteric mechanisms.

## Introduction

The classical two-state conformational model proposed by Monod and co-workers to explain allosteric interactions in hemoglobin [1], [2] has been expanded in recent years as a more generalized mechanism of protein regulation than proposed originally. Major extensions to the theory include allosteric regulation of monomeric proteins and the not necessary presence of distinctive conformational changes as a prerequisite for this type of regulation [3], [4].

Cysteine cathepsins belong to the papain-like peptidases and constitute one of the major groups of lysosomal enzymes. They are involved in diverse physiological and pathological processes and have long been thought to be regulated primarily by macromolecular active-site directed inhibitors (recently reviewed in ref. [2]). Alternative mechanisms of regulation operate without direct involvement of the active site. Glycosaminoglycans emerged as a group of regulators with diverse effects on various cysteine cathepsins, including the archetypal papain and lysosomal cathepsin B [5], [6]. The best studied example of this mode of regulation is cathepsin K, the principal peptidase in bone turnover and a promising target for the treatment of osteoporosis (reviewed in ref. [7]). Its unique collagenolytic activity was shown to be potentiated by cartilage-resident glycosaminoglycans, foremost chondroitin sulfate [8], [9]. Together with the crystal structure of chondroitin sulfate bound to cathepsin K outside of the active site [10], chondroitin sulfate and other sulfated glycosaminoglycans have been characterized as the first allosteric regulators of a papain-like peptidase [11].

Recently, we described the first low-molecular mass allosteric modifier of cathepsin K identified by *in* *silico* screening of compound libraries [12] targeted at predicted allosteric sites that are connected to the active site via an evolutionarily conserved network of residues, called a protein sector [13]. Here, we expand the characterization of cathepsin K control by describing eight further compounds that affect enzyme activity by mechanisms that, kinetically, correspond to allosteric interactions. We extensively characterize the described compounds and compare their relative effects on cathepsin K by representing their activity by a novel approach termed enzyme activity modification space.

## Results

### Identification of modifiers

**Figure 1:**
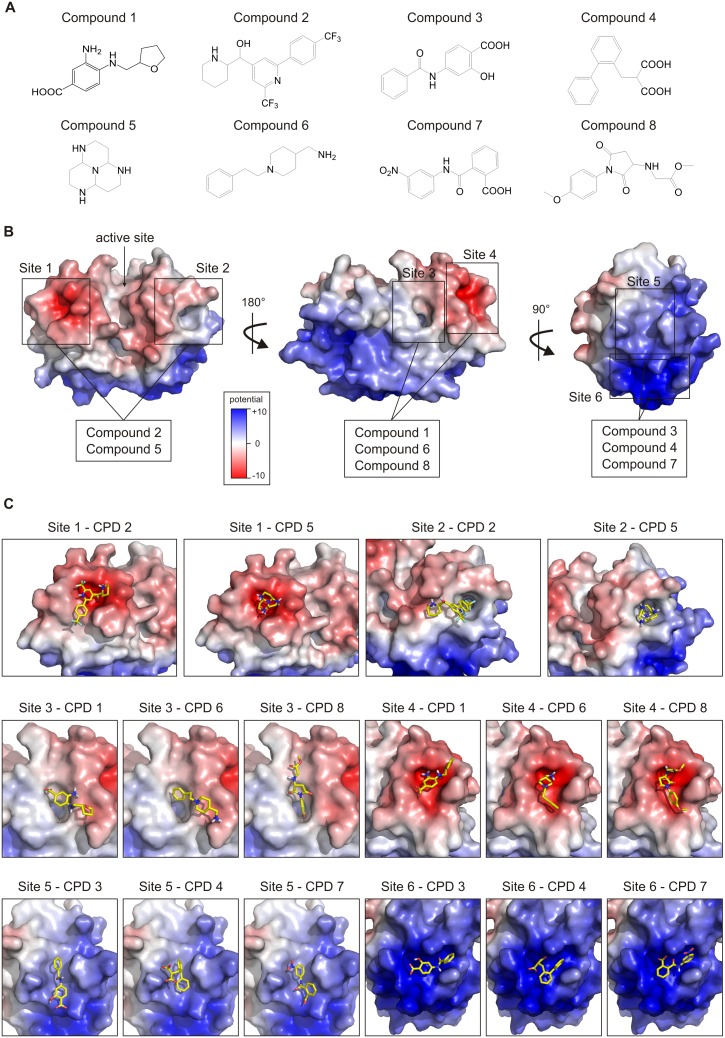
Structures, predicted binding sites and docked poses of the identified modifiers. (A) Structural formulas of the identified modifiers. (B) The locations of six potential allosteric sites on cathepsin K targeted in this study. Binding sites for each modifier marked. For reference, the location of the active site is marked by an arrow. (C) Binding poses of identified binders at their target sites on cathepsin K. In both panels, the receptor is shown in surface representation and colored according to the electrostatic potential (the colorbar is shown in the inset in panel A and is given in dimensionless units calculated by APBS) and the compounds are shown as sticks. The shown binding poses were calculated with AutoDock. Images was created with PyMOL (Schrödinger, Inc., Portland, OR, USA). Abbreviation: CPD – compound.

The prediction of seven putative allosteric sites on cathepsin K and targeting of these sites have been described previously [12]. Here, the same procedure was used to identify further allosteric modifiers. Each predicted site was targeted *in* *silico*, as described in more detail in the Materials and Methods section. The approach included a first round of high-throughput docking of compound libraries using UCSF Dock 6, followed by a second round in which the top 10% of hits were re-docked to the same site with AutoDock. Altogether, over 200 compounds were selected for experimental evaluation. Eight novel modifiers of cathepsin K are described herein. Their structures are shown in Figure 1A and further information on their identity and origin is given in Table 1. Each compound was predicted to bind to two of six different sites on cathepsin K (Figure 1B). The lowest energy binding modes calculated with AutoDock are shown in Figure 1C and the corresponding binding energies and equilibrium dissociation constants are collected in Table 2. The seventh putative allosteric site on the bottom of the molecule is not shown in Figure 1B since none of the compounds were predicted to bind there.

**Table 1:** Modifiers used in this work.

Identifier	IUPAC name	Supplier (Cat. No.)
Compound 1	3-Amino-4-[(tetrahydro-2-furanylmethyl)amino] benzoic acid	Chembridge Corp. (7607879)
Compound 2	Piperidin-2-yl-[2-(trifluoromethyl)-6-[4-(trifluoromethyl)phenyl]pyridin-4-yl] methanol	The NCI/DTP Open Chemical Repository (NSC305798)
Compound 3	N-Benzoyl-p-aminosalicylic acid	The NCI/DTP Open Chemical Repository (NSC159686)
Compound 4	(2-Biphenylylmethyl) malonic acid	The NCI/DTP Open Chemical Repository (NSC94914)
Compound 5	1, 4, 7, 9B-Tetraazaphenalene	The NCI/DTP Open Chemical Repository (NSC81462)
Compound 6	1-[1-(2-Phenylethyl)-4-piperidinyl]-methanamine	Chembridge Corp. (4009651)
Compound 7	2-[(3-Nitrophenyl)carbamoyl]benzoic acid	The NCI/DTP Open Chemical Repository (NSC408860)
Compound 8	Methyl N-[1-(4-methoxyphenyl)-2,5-dioxopyrrolidin-3-yl]glycinate	Chembridge Corp. (5855661)

**Table 2:** Binding energies and equilibrium dissociation constants calculated by AutoDock.

Modifier	Target site^1^	Estimated free energy of binding^2^ (kcal/mol)	Estimated equilibrium dissociation constant^3^ (µM)
Compound 1	Site 3	−5.98	42
	Site 4	−8.24	0.9
Compound 2	Site 1	−6.44	19
	Site 2	−5.52	90
Compound 3	Site 5	−6.73	12
	Site 6	−5.49	95
Compound 4	Site 5	−6.42	20
	Site 6	−6.33	23
Compound 5	Site 1	−8.59	0.5
	Site 2	−5.95	43
Compound 6	Site 3	−6.78	11
	Site 4	−7.18	5.0
Compound 7	Site 5	−7.52	3.0
	Site 6	−8.05	1.3
Compound 8	Site 3	−6.28	25
	Site 4	−4.33	670

1 All sites are number according to Figure 1B.

2 Corresponding to the lowest energy binding modes shown in Figure 1C.

3 Output by AutoDock as estimated “inhibition” constant calculated from the estimated free energy of binding at 298.15 K.

Cathepsin K is known for its high positive charge at about half of its molecular surface (Figure 1B) and electrostatic interactions are key to its regulation by glycosaminoglycans [10]. Charge-charge interactions are apparently also important in small molecule binding, as each of the identified modifiers contains at least one ionizable group (Figure 1A). The predicted binding sites are diverse both in shape and electrostatic potential. Whereas sites 5 and 6 are located in the positively charged area, sites 1 and 4 represent negatively charged patches on the protein’s surface and the potential of sites 2 and 3 is near-neutral. Sites 1 and 2 were each bound by compounds 2 and 5 which both contain amino groups that can interact favorably with the slightly negative or near-neutral electrostatic potential of these sites. Sites 3 and 4 were bound by compounds 1, 6 and 8. At site 4, interactions were again driven by electrostatic interactions between the negatively charged enzyme surface and amino groups of the ligands. Interestingly, site 3 has the shape of a deep narrow groove and thus appears as a typical small molecule-binding site. However, none of the predicted compounds bound within the narrow groove. Instead, interactions are limited to the region surrounding the cleft. Finally, sites 5 and 6 were bound by compounds 3, 4 and 7 which contain carboxyl groups that interact with positively charged surface residues on the enzyme. These two sites were also predicted previously as binding sites for the allosteric modifier NSC13345 and site 6 was revealed as the true binding site [12].

### Determination of kinetic mechanisms

**Figure 2:**
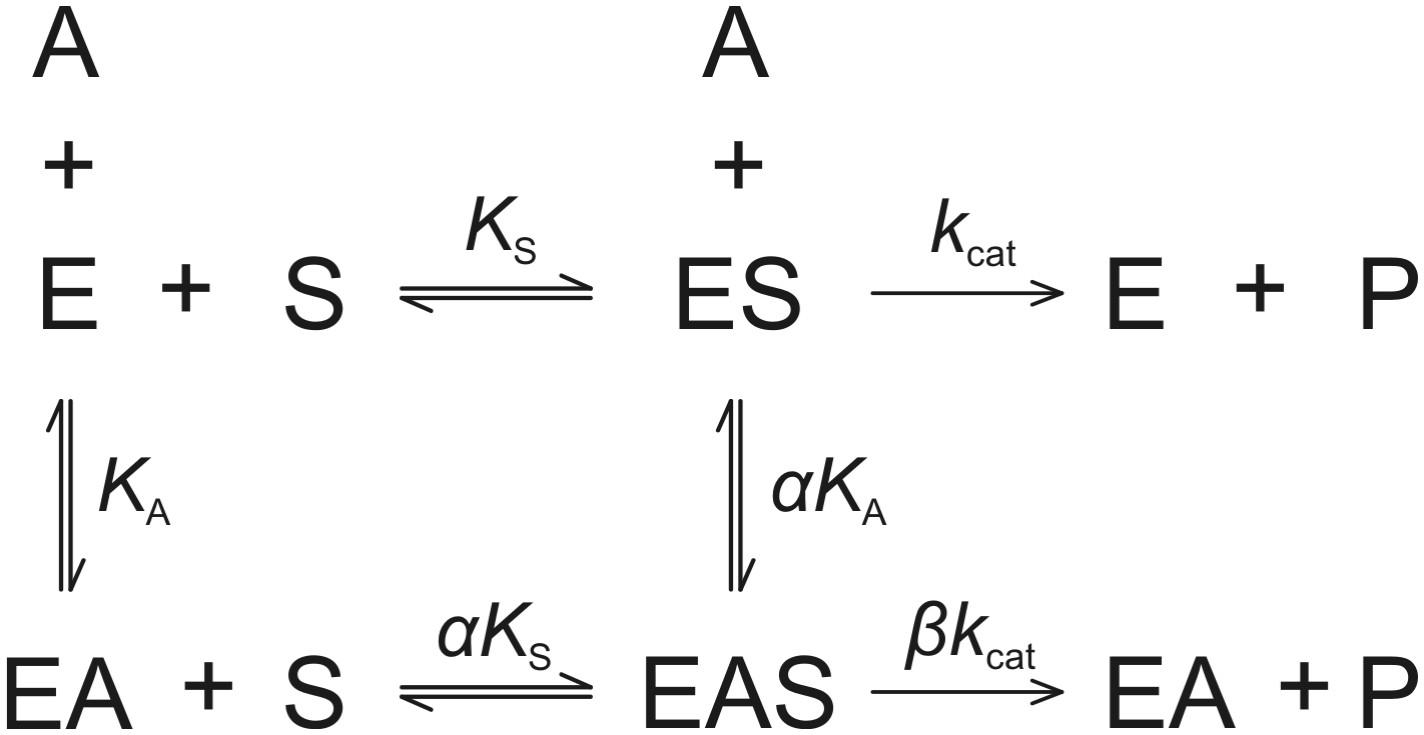
Reaction scheme for the general modifier mechanism. The scheme describes the interaction of enzyme (E) with substrate (S) and modifier (A). *K*_S_ and *K*_A_ are equilibrium dissociation constants of the ES and EA complexes under rapid equilibrium conditions, *k*_cat_ is the catalytic constant and *α* and *β* are dimensionless coefficients that determine the effect of modifier on the substrate-binding affinity of the enzyme and the catalytic rate, respectively.

All modifiers were tested for their effect on the hydrolysis of the low molecular mass synthetic substrate Z-FR↓AMC. Since all progress curves were linear from the beginning of the reaction, initial velocities were calculated from the slopes by linear regression. No significant deviations from linearity were observed for the first 120 s of the reaction and no substrate inhibition was apparent at the substrate concentrations used in this study. The basic mechanism considered for the analysis of the interactions was the general modifier mechanism shown in Figure 2, in which the effect of modifier is determined by the values of the coefficients *α* and *β* that multiply the kinetic parameters *K*_S_, *K*_A_ (*α*) and *k*_cat_ (*β*) [14]. In the thermodynamic box embodied in Figure 2, the reciprocal of the coefficient *α* represents the allosteric coupling constant that characterizes energy linkage between the modifier and the substrate [15], while a non-zero value of *β* represents the effect exerted by the modifier on catalysis. The specific velocity plot [16] was used for the primary screening of the kinetic behavior of the modifiers (Equation 2), as it allows an immediate judgment of the type of interaction and to gather preliminary estimates of the coefficients *α* and *β*, as well as of the dissociation constant of the modifier. Refinement was then performed by fitting the rate Equation 1 to data.

**Figure 3:**
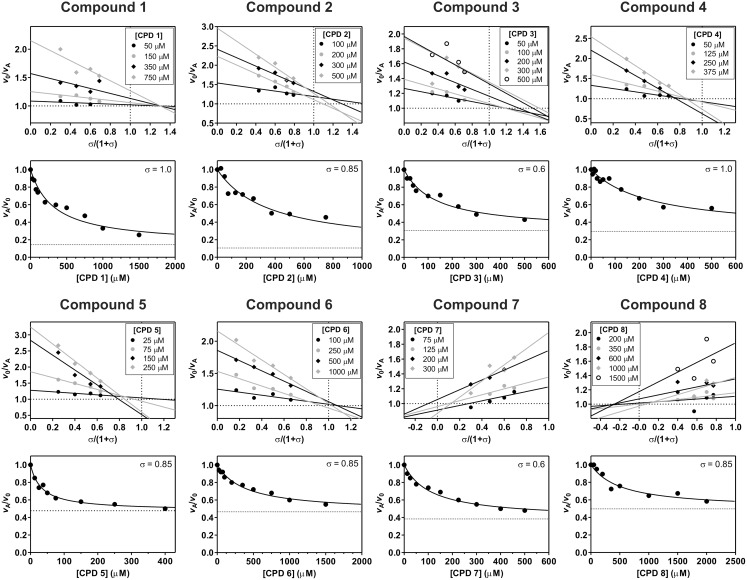
Kinetic mechanisms of the identified modifiers. All compounds were characterized by a combination of specific velocity plots (upper panels) and titration curves recorded at fixed substrate concentrations (lower panels). The kinetic parameters calculated from the combination of both methods are given in Table 3. All experiments were performed at 25±1°C in 100 mM sodium acetate buffer pH 5.50 containing 1 mM EDTA and 2.5 mM DTT using the fluorogenic substrate Z-FR↓AMC and at a final enzyme concentration of 0.5 nM.

Specific velocity plots for all modifiers are shown in Figure 3 along with relative velocity versus modifier concentration curves recorded at fixed substrate concentrations. The kinetic parameters calculated from the combinations of both methods are collected in Table 3, together with activity parameters determined from other assays described in the next section. The calculated equilibrium dissociation constants of the enzyme/modifier complex *K*_A_ were in the 10^−5^ to 10^−3^ molar range, which was expected considering the size of the compounds and the fact that they were selected without optimization of the binding geometry. All eight modifiers acted by a hyperbolic mechanism, i.e. they caused a reduction of the reaction rate without driving it to zero at saturation. They can be subdivided into four groups according to their mechanisms of modification as determined from combinations of the coefficients *α* and *β* in Figure 2. Compounds 1 through 3 were assigned to a first group with characteristic values of *α*>1 and *β*<1, which can thus be classified as hyperbolic mixed, predominantly competitive inhibitors. Compounds 1 and 2 had large values of *α*, indicating a strong competitive component, whereas that of compound 3 was only slightly above 1, a property that accounts for the different behavior of compound 3 with respect to compounds 1 and 2 in assays described in the following subsections. The second group comprised the pair of compounds 4 and 5, which belong to a class of hyperbolic mixed modifiers with a dual behavior as inhibitors or activators depending on substrate concentration and characterized by values of *α* and *β* both larger than 1 and *α*>*β*. This rare mechanism, observed previously in the case of compound NSC13345 [12], is characterized by a critical substrate concentration, which in terms of the [S]/*K*_m_ ratio *σ*, can be defined as *σ* ^0^ = (β–α)/(1–β). Actually, this calculation is based on the [S]/*K*_s_ ratio that, in virtue of the quasi-equilibrium assumption made with the use of the specific velocity plot, is approximated to [S]/*K*_m_. At substrate concentrations that give [S]/*K*_m_ smaller than *σ* ^0^ these compounds behave as inhibitors, while at substrate concentrations giving [S]/*K*_m_ larger than *σ* ^0^ they behave as activators. If the substrate concentration is such that [S]/*K*_m_ = *σ* ^0^, no effect is observed. Compound 6, which is the only representative of the third group, behaved as a hyperbolic competitive inhibitor in which the ESA complex is turned over with the same catalytic constant as the ES complex (*β* = 1). The fourth group contains a pair of hyperbolic mixed inhibitors with uncompetitive character, compounds 7 and 8, which differ from the other groups for their values of *α* and *β* being less than 1. Kinetic experiments with these two compounds were the most erratic in the series investigated. With *α*>*β* the mechanism is classified as hyperbolic mixed, predominantly uncompetitive inhibition, while with *α* = *β* the mechanism is called hyperbolic uncompetitive inhibition. In the former case, the sheaf of straight lines in the specific velocity plot should intersect the ordinate at 1 and the abscissa at a negative value. In the second case, the lines should intersect at ordinate 1 and abscissa 0. The best-fit results from the relative velocity versus modifier concentration plots shown in Figure 3 point to *α* = *β*. It remains established that, for this type of modifiers, inhibitory efficiency increases with increasing substrate concentration.

**Table 3:** Kinetic and other activity parameters of the identified modifiers.

Substrate	Z-FR↓AMC			Azocasein	Calf skin collagen
	*α* 1	*β* 1	*K*_A_ (µM)	Residual activity (in %)	*IC*_50_ (µM)^2^
Compound 1	6.7±3.0	0.55±0.25	190±20	74±9	1000
Compound 2	7.2±0.5	0.74±0.13	150±50	93±7	500
Compound 3	1.7±0.5	0.44±0.22	110±20	86±4	n.i.
Compound 4	5.6±1.2	1.5±0.3	100±40	90±3	n.i.
Compound 5	4.8±1.5	1.5±0.2	20±5	86±4	n.i.
Compound 6	3.0±0.2	1	270±50	77±8	n.i.
Compound 7	0.19±0.03	0.19±0.03	290±20	104±5	n.i.
Compound 8	0.31±0.06	0.31±0.06	1100±200	101±3	n.i.

1 Coefficients *α* and *β* multiply the kinetic parameters *K*_S_, *K*_A_ (*α*) and *k*_cat_ (*β*) and thus quantify the effect of modifier on the affinity for the substrate (*α*) and the catalytic rate (*β*), respectively.

2 n.i. indicates no inhibition.

### Inhibition of macromolecular substrate hydrolysis

**Figure 4:**
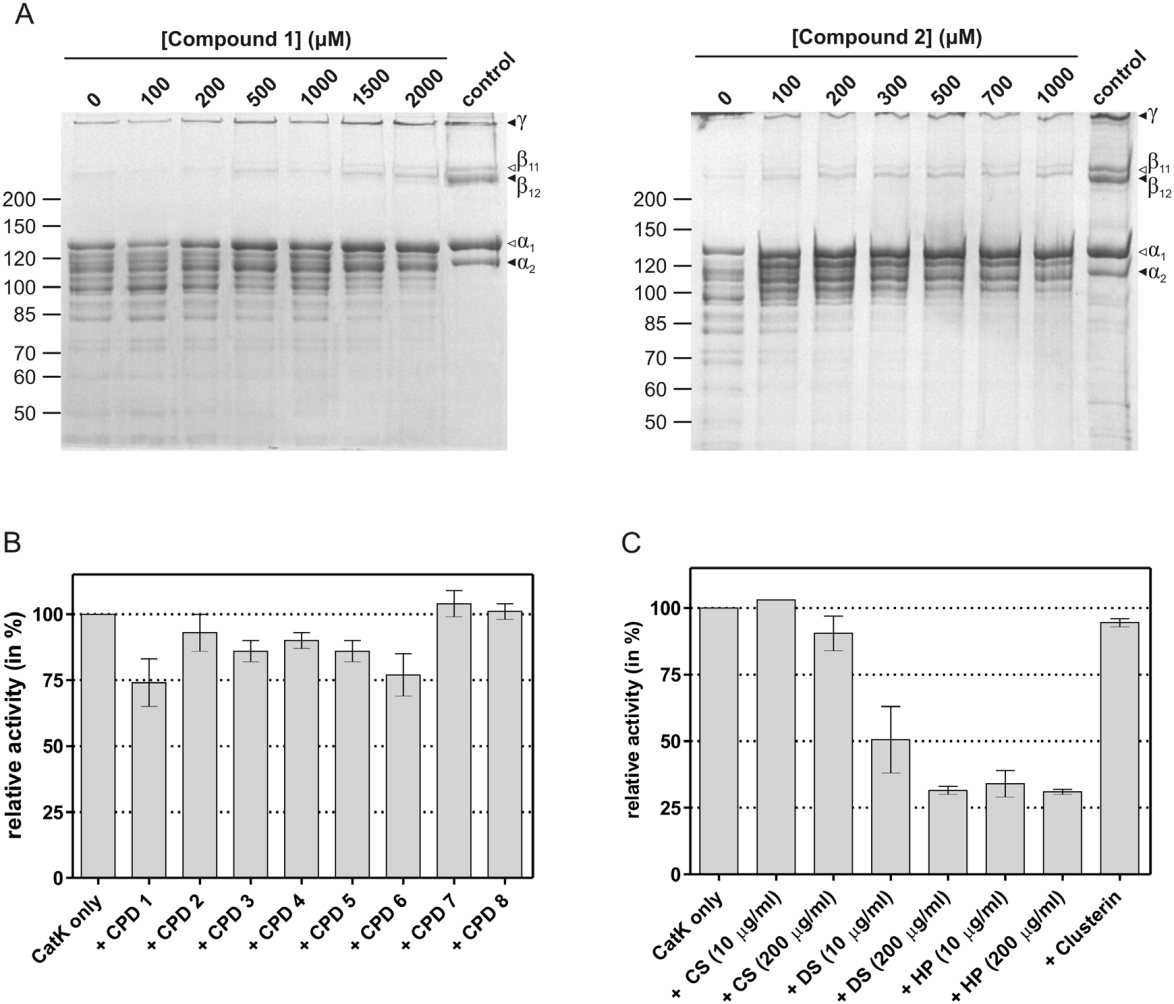
Effect of modifiers on the degradation of macromolecular substrates. (A) Effect of increasing concentrations of compounds 1 and 2 on the degradation of calf skin collagen. Molecular mass standards are given in kDa and positions of α, β and γ chains of intact collagen are marked by arrows. (B) Effect of modifiers on azocasein degradation. (C) Effect of glycosaminoglycans and clusterin on azocasein degradation. Two different concentrations of each glycosaminoglycan were used in the experiments (10 µg/ml and 200 µg/ml). Error bars in panels B and C represent the SEM of three experiments. All experiments were performed in 100 mM sodium acetate buffer pH 5.50 containing 1 mM EDTA and 5 mM DTT, as described in detail in the Materials and Methods section. Abbreviations: CPD – compound, CS – chondroitin sulfate, DS – dermatan sulfate, HP – heparin.

The use of synthetic substrates is practical for the determination of the mechanisms of modification. However, these results do not necessarily represent the effect of modifiers on the hydrolysis of macromolecular substrates. Therefore, all modifiers were also tested with two macromolecular substrates, calf skin collagen and azocasein. The collagenolytic activity was tested quantitatively by following the fragmentation of the characteristic type I collagen pattern into smaller products on SDS-PAGE after incubation of the samples with cathepsin K in the presence and absence of modifiers. Of the eight modifiers, only compounds 1 and 2 showed a concentration-dependent inhibitory effect (Figure 4A), whereas no inhibitory, or activating, activity was observed for the remaining six compounds. *IC*_50_ values were estimated at around 1 mM and 0.5 mM for compounds 1 and 2, respectively. Similarly, all compounds showed little or no inhibitory activity in azocasein degradation assays (Figure 4B). Compounds 1 through 6 reduced the activity of cathepsin K by less than 25 percent with compounds 1 and 6 being the most effective, while compounds 7 and 8 were ineffective. Due to the generally small inhibitory effects, *IC*_50_ values could be reliably determined only for the two most effective compounds. The calculated *IC*_50_ value for compound 1 was 730±190 µM and was thus comparable to the value estimated for the degradation of collagen. The calculated *IC*_50_ value for compound 6 was 1100±240 µM. Thus, *IC*_50_ was about 4-fold higher than *K*_A_ for both compounds.

For the needs of the activity space analysis presented in one of the following subsections, azocasein degradation assays were also performed in the presence of glycosaminoglycans and clusterin (Figure 4C). Two different concentrations of glycosaminoglycans were used (10 µg/ml and 200 µg/ml, respectively) to take into account the varying concentration-dependent effects of glycosaminoglycans in collagen degradation assays [17]. In all cases, glycosaminoglycans behaved as inhibitors, except the lowest concentration of chondroitin sulfate was ineffective. A marginal inhibitory effect was also observed for clusterin.

### Stabilization of cathepsin K at physiological plasma pH

Apart from directly affecting the activity of their target, allosteric modifiers have the potential to affect other aspects of enzymatic activity, such as its stability under unfavorable conditions. In the case of cathepsin K, heparin and the extracellular chaperone clusterin have been shown to increase the stability of the otherwise unstable peptidase at the physiological plasma pH value of 7.4 [11], [18]. The instability of cathepsin K has been shown to be due at least partly to autoproteolytic degradation [11]. Therefore, allosteric modifiers can contribute to the stabilization of the enzyme by at least two mechanisms, i.e. by inhibiting its proteolytic activity or by shielding the susceptible regions of the molecule from proteolytic attack by directly binding to these regions.

Here we have examined the newly identified modifiers for their effect on the stability of cathepsin K. As shown in Table 4, compounds 1, 2 and 5 increased the stability of cathepsin K by about 2-fold, which is similar to the activation effect produced by clusterin [18]. The remaining compounds were less efficient. For the needs of the activity modification space analysis, the experiment was also performed with the previously described allosteric modifier NSC1335. This compound showed a weak stabilization effect, increasing the half-life of cathepsin K by 1.2-fold. Altogether, no clear correlation between their stabilizing effect and other kinetic or activity parameters can be determined for the modifiers. It is hence likely that their overall effects on enzyme stability are due to a combination of multiple factors, as discussed above.

**Table 4:** Effect of modifiers on the stability of cathepsin K at pH 7.4.

Modifier	Half-life (min)
enzyme only	6.4±1.2
Compound 1	13.5±2.6
Compound 2	14.3±0.7
Compound 3	8.6±0.9
Compound 4	9.5±0.8
Compound 5	13.6±0.7
Compound 6	7.5±1.1
Compound 7	10.1±1.8
Compound 8	6.5±1.0
NSC13345	7.7±0.7

The enzyme was incubated at 37°C in 50 mM Hepes buffer pH 7.40 containing 1 mM EDTA and 5 mM DTT in the presence or absence of each modifier. Aliquots were withdrawn from the reaction mixtures at regular time intervals and their residual activity measured using the substrate Z-FR↓AMC.

### The activity modification space of cathepsin K

The enzyme activity modification space is a straightforward method to combine results from different experiments and globally compare the effects of different modifiers on an enzyme. We define it as an *n*-dimensional space, where *n* is equal to the number of different experimentally determined parameters presented in the particular representation. Parameters can be plotted either in terms of thermodynamic constants, values of coefficients such as *α* and *β* in the general modifier mechanism, or relative activities. Depending on the number of dimensions, data can be plotted directly or the dimensions reduced by calculating the principal components of the activity space matrix and plotting the first two or three components. In general, the activity modification space can be viewed as a part of the complete enzyme activity space which represents all possible activity profiles of a particular enzyme, caused not only by modifiers which bind the enzyme in a defined manner, but also by other environmental parameters, such as ionic strength, pH, temperature, etc.

**Figure 5:**
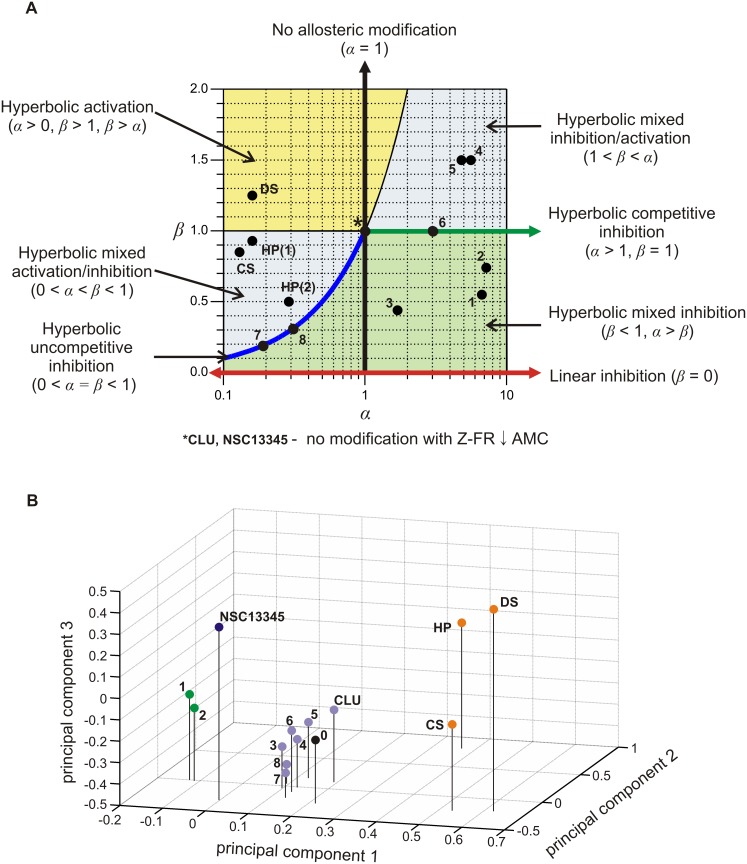
Activity modification space of cathepsin K. (A) The repertoire of binding affinities (parameter *α*) and catalytic rates (parameter *β*) with Z-FR↓AMC as substrate. Areas of the modification space that represent distinctive modes of modification are marked accordingly. Since heparin (HP) binds to two sites on cathepsin K, data for each site are presented separately in this representation. The image was created with GraphPad Prism 5.0. (B) The global effect of all known modifiers of cathepsin K other than active site-directed competitive inhibitors was analyzed with principal component analysis. Data is presented as a three-dimensional diagram of the first three principal components, which account for more than 90% of the variance. The analysis was performed on an activity modification matrix containing data collected from all four types of assays described herein (Table S1). Vertical lines in the diagram indicate the positions of each modifier in the plane defined by principal components 1 and 2. Color-coding corresponds to the division of the modifiers into different groups, based on the results of this analysis. Modifiers identified in this study are marked with consecutively increasing Arabic numerals according to Table 1. The position of cathepsin K alone is marked by “0” The image was created with Matlab. Abbreviations: CS – chondroitin sulfate, DS – dermatan sulfate, HP – heparin, CLU – clusterin.

Here the activity modification space concept is used in two independent ways to investigate the effects of all known modifiers of cathepsin K other than active-site directed inhibitors. Data for previously known modifiers was taken either from published sources or from experiments performed specifically for this purpose, as described in the previous subsections. First, a simple two-dimensional representation was constructed only from the values of the kinetic coefficients *α* and *β* determined for the modifiers when using the synthetic substrate Z-FR↓AMC (Figure 5A), the most commonly used substrate for measuring the activity of cathepsin K and other cysteine cathepsin endopeptidases *in* *vitro*. This representation shows that cathepsin K can be affected by all types of hyperbolic mechanisms determined by the general modifier mechanism, including inhibition, activation and mixed inhibition/activation mechanisms, as discussed in more detail above. The distinctively colored parts of the *α*/*β* diagram represent the combinations of both coefficients that produce different types of allosteric modification, i.e. mixed inhibition, mixed inhibition/activation or non-essential activation. Hyperbolic competitive inhibitors are defined by a combination of *α*>1 and *β* = 1, whereas uncompetitive inhibitors are defined by the combination 0<*α* = *β*<1. By definition, no allosteric modification is possible at *α* = 1 due to the absence of thermodynamic coupling between modifier and substrate [15]. A special case is represented by the point (1,1) where both coefficients *α* and *β* are equal to 1. A modifier with this combination of parameters binds to the enzyme without any measurable effect on its activity under the particular experimental conditions. One example is the recently described compound NSC13345. While the compound had no significant effect on the hydrolysis of Z-FR↓AMC, other experiments have clearly shown that the compound binds and inhibits the enzyme [12]. Another class of compounds with this particular combination of coefficients are so-called liberators, represented by the recently described clusterin [18]. Again, no effect on enzymatic activity is observed in the ternary ESA complex. However, in the presence of an inhibitor, the liberator negates the effect of the inhibitor and thus “liberates” the enzyme [19]. The representation in Figure 5A also takes into account all linear mechanisms of inhibition which lie on the abscissa (*β* = 0). They are discriminated among each other by their respective values of the coefficient *α*. For linear competitive inhibitors the value of *α* approaches ∞. Despite their absence from the representation in Figure 5A a number of such inhibitors have been described for cathepsin K, including endogenous macromolecular inhibitors as well as a plethora of active site-directed reversible inhibitors designed for cathepsin K targeting in medical applications (reviewed in ref. [7]). For linear uncompetitive inhibitors, 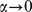, while linear mixed inhibitors are characterized by values of *α*>1. Finally, the mostly theoretical non-competitive mechanism would be characterized by the value of *α* = 1 and *β* = 0.

The approach in Figure 5A summarizes the kinetic parameters and thereby aids in identifying and categorizing the kinetic mechanisms by which cathepsin K activity can be modified. It is, however, suitable only for data obtained with synthetic substrates, which allow for the determination of the individual coefficients. To evaluate comprehensively the effect of the modifiers on cathepsin K we have utilized four different assays described above, where each assay investigates a different aspect of modifier activity. The overall potency of the modifiers, and their categorization, rely on the combined effects of each modifier across all performed assays. For a global analysis, data from all four experiments was collected in the form of an activity modification matrix (Table S1) and analyzed by principal component analysis [20]. A plot of the first three principal components which account for more than 90% of the variance are shown in Figure 5B. In general, the distance of each modifier from the position of enzyme alone can be interpreted as a measure of the strength of modification. Analogously, characteristic groups formed in the activity modification space can be interpreted as distinctive modes of enzyme modification. The modifiers investigated in the present study are divided into two groups. The first group is formed by the pair of compounds 1 and 2, which act as inhibitors in all performed assays and stabilize the enzyme at neutral pH. The remaining compounds form a loose group located in the vicinity of unmodified cathepsin K, reflecting their overall weak effect on cathepsin K. Of the previously known modifiers, NSC13345 and glycosaminoglycans, each form distinctive groups in the activity modification space distant from the position of unmodified enzyme. Not surprisingly, a significant degree of dispersion is observed in the glycosaminoglycan group (chondroitin sulfate, dermatan sulfate and heparin), resulting from their diverse effects on cathepsin K described herein and previously [11]. Last, clusterin is positioned close to unmodified cathepsin K due to its lack of effect on enzyme activity and can be considered part of the cluster formed by compounds 3 through 8.

## Discussion

The primary goal of this study was to investigate the range of effects that can be produced by allosteric modifiers of cathepsin K. The theory of enzyme kinetics does not set precise limitations in terms of the effects on substrate binding affinity or catalytic rate of any enzyme. However, the activity of enzymes is restricted by their structural limitations meaning that each enzyme can exhibit only a limited range of kinetic profiles, which can result either from its intrinsic flexibility or from the binding of an allosteric modifier. The concept of activity modification space was introduced to globally analyze the effect of the identified modifiers on cathepsin K. This method is similar to the concepts of ligand-activity space (or substrate-activity space) which uses a multidimensional representation to analyze the directed evolution of enzymes using multiple different substrates or ligands (see ref. [21] and refs. therein) and chemical space of inhibitors which was recently used to investigate the selectivity of PARP inhibitors [22].

Results obtained with the eight modifiers described in this paper and previous findings show that cathepsin K has a broad activity space and that it is very sensitive to modification by specific mechanisms, such as binding of modifier, as well as non-specific mechanisms, such as variations in the ionic strength of the medium [11]. At the structural level, this is probably related to a high structural flexibility of the molecule, which can be easily explained by its broad specificity for macromolecular proteinaceous substrates. The analysis of the activity modification space (Figure 5) identified several functionally distinct groups of compounds, indicating the presence of multiple allosteric mechanisms in the molecule. Glycosaminoglycans and NSC13345 each formed clearly distinguishable groups within the activity space related to their distinctive allosteric mechanisms of action, which have been characterized both structurally and functionally [10], [11], [12]. The newly identified modifiers are separated from these two groups indicating that they are functionally distinct. These compounds either bind to allosteric sites elsewhere on the molecule or they elicit different allosteric responses by binding to the same site on the enzyme. The latter was observed in several systems, e.g. the conformational changes associated with GTP and GDP binding to G proteins such as Ras and cAMP-dependent protein kinase (discussed in ref. [23]). The compounds form two groups in the activity modification space, one is formed by compounds 1 and 2 which exhibit the overall strongest performance in activity and stability assays, whereas the remaining compounds form a group that is closely located near unmodified cathepsin K, reflecting their overall weak activity. Given the diverse kinetic profiles and structural diversity of the latter group, these compounds are likely to bind to several different binding sites that do not efficiently transmit allosteric signals through the molecule. A comparison of computationally predicted and experimentally determined equilibrium constants (Tables 2 and 3, respectively) shows that for all compounds except 6 and 7 at least one of the predicted sites remains a viable candidate for the true binding site. Minor differences between both values can be easily accounted for by receptor flexibility, as observed in the case of NSC13345 where the experimentally determined binding constant was 5-fold lower than the computationally predicted value [12]. While the compounds 1 and 2 are commendable of further development for drug design, the remaining six are interesting not only for their *in* *vitro* kinetic effects, but also for the development of specific probes to follow cathepsin K activity *in* *vivo*. Currently available probes bind into the active site of their targets and thereby abolish the target’s activity, binding at a distant site would, however, allow to follow the enzyme in action.

In general, the distinguishable groups in the activity space identify several trends of activity modification. The inhibitory effect of NSC13345 scales proportionally with substrate size, ranging from no interaction with Z-FR↓AMC to full inhibition with collagen [12], whereas the inverse trend is observed for compounds 3 through 6. Glycosaminoglycans and compounds 1 and 2 show no clear trends in this respect, however, modifier activity is again dependent on the substrate. Finally, clusterin provides additional diversity by acting as a liberator without affecting enzyme activity *per se*. While the plethora of different effects may seem erratic, it is a clear indication that allosteric mechanisms serve to fine-tune the activity of papain-like peptidases in nature, while emergency inhibition is reserved for macromolecular inhibitors.

## Materials and Methods

### Materials

Recombinant human cathepsin K was prepared as described previously [24]. Chemical probes were obtained from the U.S. NCI/DTP Open Chemical Repository or from ChemBridge (USA). Information on their identity and origin is given in Table 1. All compounds were soluble in aqueous buffers at concentrations of at least 2 mM, except for compound 2, which was soluble up to a maximal concentration of 1 mM. Stock solutions of all compounds were prepared in DMSO. The fluorogenic substrate Z-FR↓AMC (benzyloxycarbonyl-Phe-Arg-7-amino-4-methylcoumarin) was from Bachem (Switzerland).

### Docking of compound libraries

Potential allosteric modifiers were predicted as described previously [12]. In short, the NCI Diversity Set III and ChemBridge Building Blocks compound libraries were obtained from the ZINC database [25] and filtered to retain only molecules with molecular masses between 150 and 500 Da and less than seven rotatable bonds. Both libraries were docked to all seven predicted allosteric sites on cathepsin K [12]. Cathepsin K coordinates were retrieved from the Protein Data Bank (PDB ID 1ATK). Docking was first performed with the UCSF Dock 6.4 program suite (available online at http://dock.compbio.ucsf.edu/) using default values of all parameters. The best hits (about top 10%) were selected based on the binding energies calculated by the internal scoring function and re-docked to the receptor using AutoDock 4.2 [26]. The best binders were again selected on the basis of the binding affinities calculated by the AutoDock internal scoring function. Each ligand was parameterized to allow the maximum number of rotatable bonds, but excluding amide and ring bonds. Docking was performed with the Lamarckian genetic algorithm. For each ligand, 20 runs were performed using a population of 300 individuals over 3000 generations, starting from random orientations and conformations. For presentation of the results, the lowest energy poses calculated by AutoDock were selected and visualized with PyMOL. The receptor surface was colored according to its electrostatic potential calculated with APBS [27].

### Kinetic measurements

All kinetic measurements were performed in 100 mM sodium acetate buffer pH 5.50 containing 1 mM EDTA and 2.5 mM dithiothreitol (DTT) in single-use acrylic cuvettes (1×1 cm) thermostatted at 25±1°C with magnetic stirring. The hydrolysis of Z-FR↓AMC was followed fluorometrically at λ_ex_ 383 nm and λ_em_ 455 nm. All tested modifiers were analyzed spectroscopically to determine their absorption properties under the conditions used in the assays. When necessary, appropriate corrections were made to the calculated reaction rates to take into account the inner filter effect [28].

### Kinetic model

The effects of the modifiers on enzymatic activity were analyzed with the general modifier mechanism [14] which describes the interaction between enzyme (E), substrate (S) and modifier (A), as shown in Figure 2. Assuming that binding of A to E and ES is at quasi-equilibrium and that steady-state conditions are established in the fluxes around ES and ESA (i.e. the catalytic steps), the reaction rate for the mechanism in Figure 2 is defined by Equation 1:
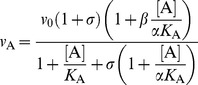
(1)where *v*_0_ is the reaction rate in the absence of modifier, *K*_A_ is the equilibrium dissociation constant of the EA complex, *α* and *β* are dimensionless coefficients and *σ* = [S]/*K*_m_, approximated to [S]/*K*_s_. To determine the mechanism of interaction, plot the kinetic data and calculate preliminary values of the interaction parameters, the specific velocity plot was used [16]. For this purpose, Equation 1 is rewritten as:



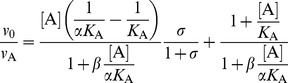
(2)


The plot of *v*_0_/*v*_A_ versus σ/(1+σ) produces straight lines with intersection points at *v*_0_/*v*_A_ = 1, regardless of the interaction mechanism. The parameters *α*, *β* and *K*_A_ are determined by replotting the extrapolated values of the straight lines at σ/(1+σ) = 0 (a) and σ/(1+σ) = 1 (b) versus 1/[A] in the form:
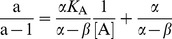
(3)

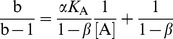
(4)


### Collagenolytic assays

Soluble calf skin collagen (Worthington Biochemical Corporation, USA) was diluted in 100 mM sodium acetate buffer pH 5.50 containing 1 mM EDTA and 5 mM DTT to a final concentration of 0.5 mg/ml. Experiments were started by addition of cathepsin K (final enzyme concentration 0.6 µM) to the reaction mixture containing collagen and increasing concentrations of modifier. All assays were performed at 28°C with shaking on an Eppendorf Thermomixer Comfort. Samples were incubated for 2 hours and the reactions then blocked by addition of E-64 to a final concentration of 1 µM. 10 µg of digested collagen were analyzed by SDS-PAGE on 8% polyacrylamide gels and protein bands visualized by Coomassie Brilliant Blue R-250 staining.

### Azocasein degradation assays

Azocasein (Sigma-Aldrich, USA) was dissolved in 100 mM sodium acetate buffer pH 5.50 containing 1 mM EDTA and 5 mM DTT at a concentration of 3 mg/ml. Samples were incubated with 0.1 µM cathepsin K for 30 min at 37°C in the presence of each compound. All compounds were used at concentrations increasing up to 2 mM, except compound 2, which was used at a maximal concentration of 1 mM due to its limited solubility at higher concentrations. Reactions were stopped by addition of trichloroacetic acid (final concentration 5% v/v), centrifuged, clear supernatants mixed with 0.5 M NaOH and the concentrations of solubilized peptides determined by measuring the absorbance at 440 nm. All experiments were performed in triplicates and appropriate blanks were run to take into account the absorption of azocasein and compounds alone. *IC*_50_ values were determined as described previously [12].

### Thermal stability of cathepsin K in the presence of modifiers

Cathepsin K was incubated at 37°C in 50 mM Hepes buffer pH 7.40 containing 1 mM EDTA and 5 mM DTT in the presence or absence of each modifier. The final enzyme concentration was 0.2 µM and final compounds concentrations were 2 mM, except compound 2, which was used at a concentration of 1 mM. All experiments were performed in Eppendorf Protein LoBind Tubes to minimize the adsorption of proteins on tube walls. Aliquots were withdrawn from the reaction mixtures at regular time intervals and their residual activity measured using the substrate Z-FR↓AMC (5 µM final concentration). Inactivation curves had exponential profiles that were fitted with the mathematical model for one-phase exponential decay to determine the half-life of the enzyme in each reaction mixture.

### Principal component analysis of the activity modification space

For analysis, the results of each experiment were presented as one data set termed the activity modification matrix (Table S1). Data from experiments performed with the synthetic substrate Z-FR↓AMC was included as the geometric mean of residual activities (*v*_A_) at a low substrate concentration (0.1×*K*_m_) and a high substrate concentration (10×*K*_m_), both in the presence of a saturating modifier concentration (10×*K*_A_). For each modifier, Equation 1 was used to calculate the appropriate values of *v*_A_ using experimentally determined values of the parameters *α* and *β*. Stabilization of the enzyme was expressed as ln(*sf*), where the stabilization factor *sf* is the ratio of half-lives determined in the presence and absence of modifier. Azocasein degradation data was input as the fraction of residual enzyme activity (a value of 1 equals the activity of unmodified enzyme). For collagenolytic assays, qualitative values of 1 and 0 were used to signify inhibition and lack thereof, respectively. For glycosaminoglycans, a value of −1 was used to signify their concentration- and environment-dependent activation/inhibition activity. Principal component calculations and graphical visualizations were performed in the Matlab environment.

## Supporting Information

Table S1 **The activity modification matrix used for principal component analysis.**
(PDF)
