# Supplementary material for: Probing the Activity Modification Space of the Cysteine Peptidase Cathepsin K with Novel Allosteric Modifiers
Source: PLoS One. 2014 Sep 3;9(9):e106642. doi: 10.1371/journal.pone.0106642 (PMC4153677; doi:10.1371/journal.pone.0106642)
Supplement: Table S1 — The activity modification matrix used for principal component analysis. (PDF) [file pone.0106642.s001.pdf]

Table S1. The activity modification matrix used for principal component analysis. The table contains a collection of data from this manuscript and from previous work, as described in the main text. Data from experiments performed with Z-FR↓AMC is included as the geometric mean of residual activities (v_A) at a low substrate concentration ($0.1 \times K_m$) and a high substrate concentration ($10 \times K_m$), both in the presence of a saturating modifier concentration ($10 \times K_A$). The calculations were performed with Equation 1 based on experimentally determined values of the kinetic coefficients α and β in Table 3. Azocasein degradation data is given as the fraction of residual enzyme activity (a value of 1 equals the activity of unmodified enzyme). For collagenolytic assays, qualitative values of 1 and 0 were used to signify inhibition and lack thereof, respectively, and a value of -1 was used to describe the concentration-dependent roles of glycosaminoglycans. Stabilization of the enzyme is expressed as ln(*sf*), where the stabilization factor *sf* is the ratio of half-lives determined in the presence and absence of modifier at 37 °C and pH 7.4. Abbreviations: CATK – cathepsin K, CPD – compound, CS – chondroitin sulfate, DS – dermatan sulfate, HP – heparin, CLU – clusterin.

	Z-FR↓AMC	azocasein	collagen	stability
CATK	1.00	1.00	0	0.00
CPD 1	0.37	0.74	1	0.74
CPD 2	0.42	0.88	1	0.79
CPD 3	0.42	0.83	0	0.26
CPD 4	0.70	0.89	0	0.41
CPD 5	0.74	0.86	0	0.74
CPD 6	0.65	0.77	0	0.18
CPD 7	0.48	1.01	0	0.47
CPD 8	0.60	1.04	0	0.02
NSC13345	1.00	0.40	1	0.18
CS	2.40	0.90	-1	-0.24
DS	3.18	0.32	-1	-0.15
HP	1.55	0.31	-1	1.69
CLU	1.00	0.95	0	0.79
